# A time-updated scoring system derived from a nomogram to predict 3-month mortality in maintenance hemodialysis patients

**DOI:** 10.3389/fragi.2026.1716325

**Published:** 2026-01-15

**Authors:** Li Cheng, Shuai Fu, Yonglong Min, Dongdong Mao, Can Tu, Qianshen Zhu, Wenhui Qiu, Hongbo Li, Fei Xiong

**Affiliations:** Department of Nephrology, Wuhan No.1 Hospital, Wuhan, China

**Keywords:** maintenance hemodialysis, mortality, nomogram, risk score, time-updated

## Abstract

**Objective:**

This study aimed to develop and validate a novel risk score for death within the next 3 months in patients with maintenance hemodialysis (MHD).

**Methods:**

All the data were derived in the Wuhan Hemodialysis Quality Control Center and were divided into the training set (2019–2021, n = 19,735) and the validation set (2022–2023, n = 15,265). The primary outcome was the all-cause mortality within 3 months after regular monthly laboratory tests. The predictive model was displayed as a nomogram and modified into a novel scoring system based on the coefficients of multivariable logistic regression.

**Results:**

There were 1684 (8.5%) patients and 1670 (10.9%) patients who died in the development and validation set, respectively. The final novel score system was calculated based on the five predictors: age ≥60 years (2 points), dialysis duration <1 year (2 points), catheter usage (1 point), hemoglobin <110 g/L (1 point), and albumin <35 g/L (3 points). This model with a C-index of 0.72 and 0.73 on the two sets and exhibited a significant ability in stratification of patients into low-risk, intermediate-risk, and high-risk groups (P < 0.0001).

**Conclusion:**

This easy-to-use applicable scoring system accurately predicts 3-month mortality in HD patients, facilitating risk stratification and personalized care.

## Introduction

Maintenance hemodialysis (MHD) has evolved as a life-sustaining therapy for patients with end-stage kidney disease (ESKD), putting a strain on medical resources each year and becoming a major public health concern, particularly in China (Cao et al., 2023; Li Z. et al., 2024; Li et al., 2025). Despite significant advances in dialysis technology and understanding, the overall death rate among MHD patients has remained unacceptably high in recent decades, which can be attributed, at least in part, to regional economic circumstances (Miyauchi et al., 2023; Zhu et al., 2024). As a result, it is critical to seek a user-friendly model as early and cheaply as feasible with precision for the early diagnosis of individuals at risk of death, which may improve monitoring methods and therefore improve the prognosis of MHD patients.

Current risk classification models for MHD patients are generally based on a set of clinical characteristics such as medical history, laboratory findings, and imaging studies (Zhou and Zhu, 2024; Yang et al., 2025; Xing et al., 2024). However, these models have demonstrated moderate predictive accuracy and may perform better when including factors that are not commonly accessible for MHD patients or using complicated prediction techniques for model creation (e.g., machine learning) (Xing et al., 2024; Al et al., 2024; Nakagawa et al., 2022). Furthermore, while many previous models focused on longer-term mortality (e.g., 3-year and 5-year), there is still significant utility in identifying the patients most likely to die in the near term (less than 1 year), due to the variability of death rates for MHD patients (Zhou et al., 2021; Kang et al., 2024). In addition, several risk scores were created and tested for the near-term mortality based on the initial laboratory results for incident HD patients, which might be at least less useful due to the time-updated and abundant electronic health record data for these patients (HD patients have to undergo routine laboratory tests every 3 months or even monthly) (Thamer et al., 2015; Park et al., 2025; Wick et al., 2017). As a result, in this work, we created a therapeutically applicable prediction model utilizing multivariate logistic regression based on routinely available and time-updated dialysis record data to continually assess the probability of near-term (3-month) death for easy implementation in diverse clinical settings in MHD patients. The core prediction model was then presented as a nomogram and transformed into a unique scoring system for simplicity of future deployment. Furthermore, we verified the improved scoring model’s predictive performance and provided metrics of accuracy and decision rule performance for predictions at various thresholds.

## Materials and methods

### Patients and study design

**FIGURE 1:**
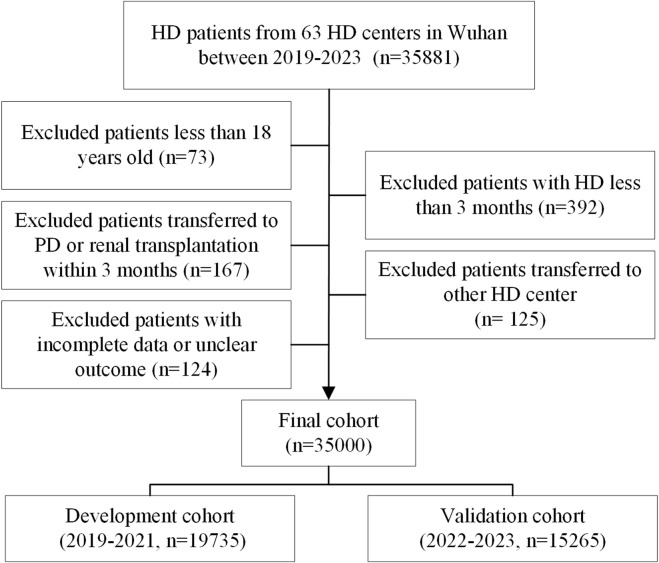
The flow chart of this study.

Data for this investigation were received from the Wuhan Hemodialysis Quality Control Center (WHQCC), which administers the majority of Wuhan’s HD facilities (63 centers). The WHQCC employed an electronic data collection system to gather patient-level data quarterly, which means that every patient had at least four times as many characteristics once a year if they did not die or were moved to peritoneal dialysis (PD) or kidney transplantation. Our research included all MHD patients listed in the WHQCC database between 1 January 2019, and 31 December 2023. Patients aged 18 or older, with a dialysis history of at least 90 days were eligible. Exclusion criteria included HD patients who were less than 18 year-old (n = 73), with HD less than 3 months (n = 392), moved to PD or kidney transplantation within 3 months (n = 167), transferred to another HD center (n = 125), or had missing or unclear data (n = 124, Figure 1). Finally, this study comprised 35,000 patients, who were separated into two groups depending on their registration year: the training set (2019–2021, n = 19,735) and the validation set (2022–2023, n = 15,265).

The Ethics Committee of Wuhan No. 1 Hospital approved this study without obtaining informed consent from the participants (No. 2025-71), considering that this was a retrospective study and all patients were de-identified or maintained with confidentiality.

### Characteristics of patients

We used characteristics that were easily available in the WHQCC database, such as demographic demographics, major causes of ESKD, dialysis duration, and laboratory findings. Furthermore, we arranged the data in a time-updated patient-quarter manner, with one row of data for each calendar quarter in which a patient attended at least one MHD session at the WHQCC. Variables were created for each patient quarter based on time-updated data. The models excluded patient quarters that happened after patients were transferred out of WHQCC care.

### Outcomes

The primary outcome was all-cause near-term mortality, which was defined as death from MHD within 3 months after the regular monthly laboratory tests. Considering that kidney transplantation is a competing event, which might prevent death on MHD, we excluded them as an accepted approach to deal with competing events (Lau et al., 2009).

### Statistics

All calculations were done with the statistical software R (version 4.1.0). Logistic regression analyses were run, and the statistically significant variables were then chosen for multivariable logistic regression analysis. To evaluate possible multicollinearity among variables, we calculated the variance inflation factor (VIF) for each variable, with a VIF larger than 10 indicating the occurrence of multicollinearity (Kim, 2019). The variable relevance of each predictor was determined by comparing the partial log probability of the logistic regression model that included or excluded all terms involving the predictor. We utilized the Wald test (χ^2^ statistic) to determine if the stated variable substantially improved model fit. The regression coefficients were used as weights for the variables in the prediction model. The scoring system’s nomogram was created using the “rms” package in R using independent risk variables from multivariate logistic analysis. A risk score for each patient was produced automatically and analyzed using a ROC curve. The final risk score was used to divide all MHD patients into three risk groups: low, middle, and high, with thresholds corresponding to clinically relevant risk differences. A two-sided P-value of less than 0.05 was considered statistically significant.

## Results

### Characteristics of patients

This study included a total of 35,000 people, who were separated into two groups based on their years of enrollment: the development set (n = 19,735) and the validation set (n = 15,265). The group was predominantly male (21,825, 62.3%), with an average age of 63.0 ± 13.6 (18–98) years. The majority of patients (n = 9889, 25.3%) were on dialysis for 1–5 years, followed by 6–10 years (n = 8543, 24.4%), with a mean HD duration of 4.9 ± 1.8 (0.3–35.1) years. Furthermore, chronic glomerulonephritis (n = 11,280, 32.2%) was the most common cause of ESKD, followed by diabetes (n = 8038, 23.0%), hypertension (n = 7135, 20.4%), and other or unidentified causes (n = 8447, 24.1%). Moreover, the 3-month mortality occurred in 3354 (9.6%) patients during this study period. Table 1 summarizes the baseline characteristics of the training set and test set.

**TABLE 1:** Baseline characteristics for the development and validation cohorts.

Characteristics	Development cohort	Validation cohort	P value
N	19,735	15,265	-
Age, years old	63.8 ± 13.7	61.9 ± 13.4	<0.001
Gender, male, n (%)	12,219 (61.9)	9606 (62.9)	0.052
Duration of dialysis, years	5.4 ± 1.9	4.4 ± 1.8	<0.001
<1 year, n (%)	1179 (6.0)	1817 (11.9)	​
1–5 years, n (%)	4554 (23.1)	5335 (34.9)	​
6–10 years, n (%)	5324 (27.0)	3219 (21.1)	​
>10 years, n (%)	2389 (12.0)	1408 (9.3)	​
Cause of ESKD, n (%)	​	​	<0.001
Chronic glomerulonephritis	6650 (33.7)	4730 (31.0)	​
Diabetic nephropathy	4412 (22.4)	3626 (23.8)	​
Hypertensive nephropathy	4038 (20.5)	3097 (20.3)	​
Others or unknown	4635 (23.4)	3812 (25.0)	​
Vascular access, n (%)	​	​	0.806
AVF	14,340 (72.7)	11,109 (72.8)	​
TCC	5395 (27.3)	4156 (27.2)	​
Laboratory results			
Hemoglobin, g/L	103.2 ± 18.3	106.5 ± 18.0	<0.001
<110 g/L, n (%)	12,614 (63.9)	8207 (53.8)	​
Platelets, × 10^9^/L	171.2 ± 55.9	171.6 ± 58.8	0.565
Albumin, g/L	38.3 ± 4.3	38.1 ± 4.3	<0.001
<35 g/L, n (%)	3831 (19.9)	3338 (21.9)	​
Potassium, mmol/L	4.7 ± 0.8	4.7 ± 0.8	0.019
Hyperpotassemia, n (%)	2573 (13.0)	2068 (13.5)	​
Calcium, mmol/L	2.2 ± 0.2	2.2 ± 0.2	<0.001
Hypercalcemia, n (%)	1769 (9.0)	1279 (8.4)	​
Hypocalcemia, n (%)	4575 (23.2)	3848 (25.2)	​
Phosphorus, mmol/L	1.7 ± 0.6	1.7 ± 0.6	<0.001
Hyperphosphatemia, n (%)	7673 (38.9)	5122 (33.6)	​
Hypophosphatemia, n (%)	1781 (9.0)	2153 (14.1)	​
iPTH, pg/mL	424.1 ± 107.7	390.5 ± 75.2	<0.001
<150 pg/mL	3409 (17.3)	3117 (20.4)	​
>600 pg/mL	3487 (17.7)	2242 (14.7)	​
Ferritin, ug/L	179.7 ± 63.0	210.9 ± 99.5	<0.001
<200 ug/L	15,360 (77.8)	10,696 (70.1)	​
Three-month death, n (%)	1684 (8.5)	1670 (10.9)	<0.001

ESKD, end-stage kidney disease; AVF, arteriovenous fistula; TCC, tunneled central venous catheter; iPTH, intact parathyroid hormone.

### The risk factors for the key outcome

To evaluate the risk factors for the primary outcome, a univariable logistic regression analysis was done, which revealed that 11 variables were coarsely linked with 3-month mortality (all variables had a VIF of less than 10, Supplementary Table S2). Following backward selection, the final multivariable logistic regression model for the primary outcome included five independent variables: age, dialysis duration, vascular access, serum hemoglobin, and albumin (Supplementary Table S3). Moreover, serum albumin less than 35 g/L, age ≥60 years old, and duration less than 1 year were identified as the best predictors of 3-month mortality (Table 2).

**TABLE 2:** A novel scoring system developed from the nomogram for 3-month death in the training cohort.

Variables	Coefficients	OR (95%CI)	Importance^a^	Score modified from coefficients
Age ≥60 years old	1.026	2.79 (2.42–3.22)	199	2
Duration <1 year	1.036	2.82 (2.42–3.29)	173	2
Vascular access	​	​	​	​
AVF	-	Ref.	​	​
TCC	0.434	1.54 (1.37–1.74)	50	1
Hemoglobin< 110 g/L g/L	0.659	1.93 (1.69–2.21)	92	1
Albumin<35 g/L	1.364	3.91 (3.49–4.39)	544	3

^a^ Defined by the χ2 Wald statistic (p < 0·001 for all variables). OR, odds ratio, 95%CI, 95% confidence interval, AVF, arteriovenous fistula; TCC, tunneled central venous catheter.

### The construction and performance of a nomogram and novel scoring system in the training set

**FIGURE 2:**
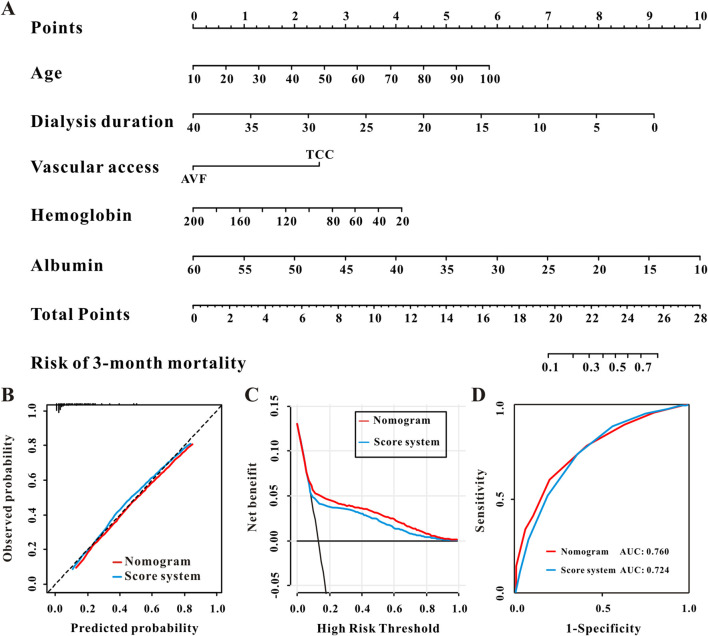
Construction and the performance of the nomogram for 3-month mortality. **(A)** The nomogram for 3-month mortality, the calidation plot **(B)**, decision curve analysis **(C)**, and the receiver operator characteristic curve **(D)** of the nomogram and the score system for 3-month mortality in the training set.

The risk of 3-month death was then calculated using a multivariate logistic regression model, resulting in a nomogram (Figure 2A). The calibration curve indicated that indicated good agreement between the predicted probabilities and the observed outcomes, which suggested that the predictions of the nomogram were accurate and not systematically over- or under-estimated (Figure 2B). The predictive nomogram’s clinical value was assessed using decision curve analysis (DCA), which showed that using the nomogram for clinical decision-making provided a higher net benefit compared to the strategies of “treating all” or “treating none” across a wide range of threshold probabilities (Figure 2C). Furthermore, this prediction nomogram had rather strong discriminative performance, as evidenced by AUC values of 0.76 (95% CI 0.75–0.78) and 0.75 (95% CI 0.74–0.76) in the validation set (Figure 2D; Table 3).

**TABLE 3:** The performance of the nomogram and the score system for the risk of 3-month mortality.

Variables	In the training cohort		In the validation cohort	
	Nomogram	Score system	Nomogram	Score system
Cutoff value	16	6	14	6
Youden index	0.408	0.386	0.382	0.360
Sensitivity (%)	79.3 (77.0–82.5)	62.1 (59.6–64.6)	73.5 (71.3–75.6)	58.1 (55.6–60.4)
Specificity (%)	72.5 (71.8–76.1)	76.5 (75.9–77.1)	64.7 (63.9–65.5)	78.0 (77.3–78.7)
PPV (%)	24.9 (23.7–26.1)	20.6 (18.8–22.1)	20.4 (19.8–21.0)	24.5 (23.5–25.4)
NPV (%)	96.1 (95.9–96.4)	94.8 (94.5–95.2)	95.2 (94.8–95.6)	93.8 (93.5–94.1)
AUC^a^	0.76 (0.75–0.78)	0.72 (0.71–0.73)	0.75 (0.74–0.76)	0.73 (0.72–0.74)

PPV, positive predictive value; NPV, negative predictive value; AUC, area under the curve.

^a^ Delong test of AUC, in the training set and the validation set were Z value = 3.817, P = 0.0001, and Z value = 3.640, P = 0.0003, respectively.

**FIGURE 3:**
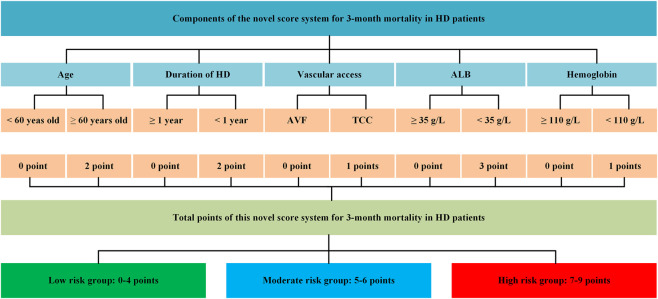
The novel score system for the risk of 3-month mortality.

To make this predictive model easier for clinicians to employ in clinical practice, we changed the nomogram to a scoring system using integer points, the point assignment was based on the relative magnitude of the regression coefficients from the multivariable logistic model, ensuring that the point values reflect the statistical weight of each predictor: serum albumin <35 g/L (3 points), age ≥60 years old (2 points), duration <1 year (2 points), hemoglobin< 110 g/L (1 point), and the vascular access was tube (1 point) (Table 2; Figure 3). Additionally, the scoring system’s numerical discriminative capacity with the nomogram was comparable, with AUCs in the training and validation sets (Delong test, P = 0.0001 and 0.0003, respectively) of 0.72 and 0.73. Additionally, calibration curves and DCA demonstrated that this scoring system was clinically useful and calibrated as the predictive nomogram (Figures 2B–D; Table 3). The validation set also showed similar results (Supplementary Figure S1A–C).

**FIGURE 4:**
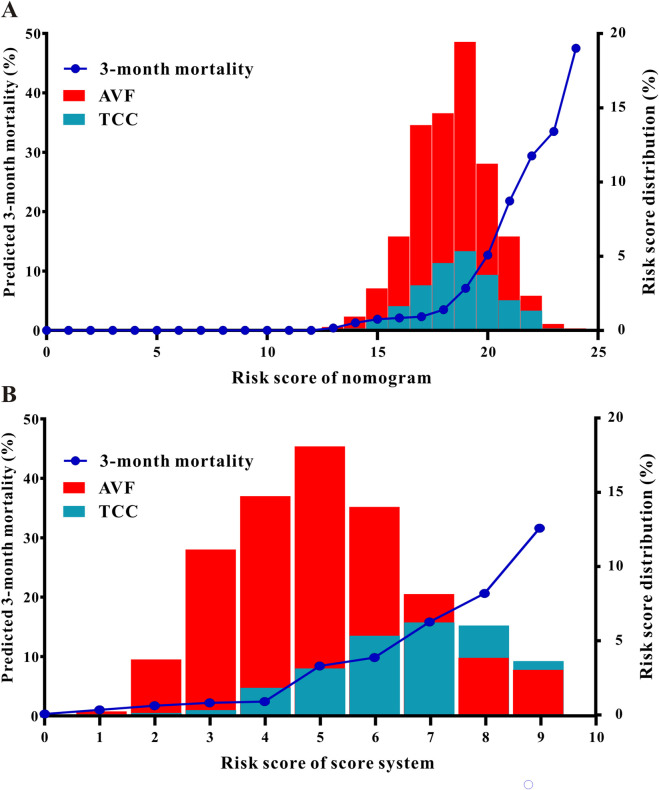
Scores calculated with the nomogram **(A)** and the score system **(B)** were plotted on the x-axis of this figure, enabling the inference of predicted 3-month mortality by correlating each score with the appropriate plotted lines. The histogram depicts the risk score distribution of the training as an example of the distribution of risk scores across the patient population (each bar represents the proportion of patients in the cohort that was assigned that specific score).

In addition, scores computed using the nomogram and the scoring system were shown in the development set (Figures 4A,B) and the validation set (Supplementary Figure S2A, B). This allowed for the correlation of each score with the relevant depicted lines, which allowed for the inference of anticipated 3-month mortality.

### Risk stratification of the risk score for 3-month mortality

**FIGURE 5:**
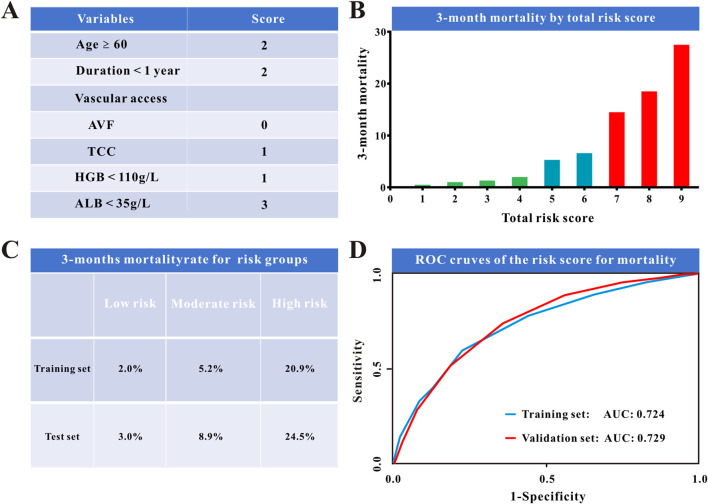
**(A)** Novel risk scoring system for the risk of 3-month mortality in MHD patients. **(B)** 3-month mortality rate by novel risk score plot. **(C)** 3-month mortality rate for the low-risk, moderate-risk, and high-risk group. **(D)** ROC curves comparing the performance of the risk model in the training and in the validation set.

In risk stratification, the scoring method did well; Figure 5A shows that higher risk scores were associated with a proportional increase in 3-month mortality. Based on their associated risks in the training set, we divided the patients into three groups: low-risk (n = 5627), intermediate-risk (n = 8735), and high-risk (n = 5373). Low risk was defined as a score of 4 or less, with 3-month death rates below 3%. With 3-month death rates considerably above 15%, scores of 7 or higher were deemed high risk. The intermediate-risk group, which had scores between 5 and 6, is shown in Figure 5B. Each risk category’s baseline characteristics are reported in Supplementary Table S3. In the training sample, the risk stratification model projected 3-month death rates of 2.0% for low-risk, 5.2% for intermediate-risk, and 20.9% for high-risk groups. Furthermore, the same results were seen in the test set (Figure 5C). The AUC for 3-month mortality for this risk score was 0.72 and 0.73 in the training and validation sets, respectively (Figure 5D). After adjusting for confounding factors, high-risk individuals had a nearly 12-fold higher 3-month mortality compared to low-risk patients (OR: 11.95; 95% CI: 9.71–14.69; P < 0.001), while moderate-risk individuals had a 2-fold higher 3-month mortality than low-risk patients (OR: 2.61; 95% CI: 2.11–3.23; P < 0.001) (Table 4).

**TABLE 4:** The association between the nomogram and the novel score for 3-month mortality.

Exposure	Model 1		Model 2		Model 3	
	OR (95%CI)	P	OR (95%CI)	P	OR (95%CI)	P
In the training set						
Score as continuous	1.71 (1.66–1.76)	<0.001	1.72 (1.66–1.78)	<0.001	1.67 (1.62–1.72)	<0.001
Score as categorical						
Low risk (1–4 points)	Ref.	-	Ref.	-	Ref.	-
Moderate risk (5–6 points)	2.70 (2.19–3.33)	<0.001	2.73 (2.21–3.37)	<0.001	2.61 (2.11–3.23)	<0.001
High risk (7–9 points)	13.13 (10.76–16.02)	<0.001	13.53 (11.08–16.53)	<0.001	11.95 (9.71–14.69)	<0.001
In the validation set						
Score as continuous	1.56 (1.50–1.60)	<0.001	1.56 (1.52–1.61)	<0.001	1.55 (1.50–1.60)	<0.001
Score as categorical						
Low risk (1–4 points)	Ref.	-	Ref.	-	Ref.	-
Moderate risk (5–6 points)	3.18 (2.65–3.82)	<0.001	3.19 (266–3.84)	<0.001	3.08 (2.56–3.71)	<0.001
High risk (7–9 points)	10.55 (8.84–12.58)	<0.001	10.77 (9.02–12.85)	<0.001	855 (7.08–10.33)	<0.001

OR, odds ratio, 95%CI, 95% confidence index, Model 1 was unadjusted, model 2 adjusted for gender and cause of ESKD., Model 3 adjusted for model 2 plus laboratory results (except for serum albumin and hemoglobin).

The model’s capacity to stratify risk independently among cohort subgroups was tested using subgroup analysis for each variable. The model worked well in the risk stratification of cohort subgroups, with full results provided in Supplementary Tables S4, S5. Moreover, this risk score had comparable performance compared with two popular risk score developed in prevous studies (Supplementary Table S5).

## Discussion

This retrospective study first developed a predictive nomogram based on easily accessible and time-updated characteristics for 3-month mortality using data from the WHQCC database of 63 MHD centers in Wuhan, and we discovered that the nomogram had adequate predictive ability and clinical utility for MHD patients. Furthermore, a simple and portable scoring system derived from the nomogram was developed for risk stratification of 3-month mortality in MHD patients. This scoring system obtained high accuracy for 3-month mortality and was verified in the validation set. To our knowledge, this is the first and most comprehensive study to design and validate a unique risk score for MHD patients using generally available criteria, with the largest sample size to date.

Despite major advances in understanding and services in recent decades, the prognosis for those with MHD has remained poor, with cardiovascular disease recognized as the leading cause of death (Cozzolino et al., 2018; He et al., 2021). As a consequence, identifying individuals who are at high risk of death early on may result in more effective therapies and, as a result, a better prognosis for MHD patients. Nonetheless, several attempts have been made to develop a more effective strategy for predicting the prognosis of MHD patients. Unfortunately, no specific approach has been used in clinical settings. Li et al. conducted a retrospective study of 285 ESKD patients and created a nomogram based on the cardiovascular calcification of thoracic computed tomography and achieved an AUC of 0.975 for 5-year mortality (Li Q. et al., 2024). Another multi-center prospective cohort of 388 Chinese MHD patients also constructed a prognostic model based on the serum fibroblast growth factor 21 and demonstrated an AUC of 0.841 (95%CI 0.795–0.887) (Li et al., 2023). In addition, a recent retrospective study established a machine learning model based on the 85 variables of 359 MHD patients and gained a sensitivity of 86% and a specificity of 75% with an AUC of 0.86 (Chen et al., 2025). Despite their substantially greater diagnosis accuracy, the aforesaid approaches could not be used in most hospitals, especially basic hospitals, due to the rigorous requirements for medical equipment or sophisticated algorithms. In other words, these procedures are not suitable for everyday clinical practice due to their discomfort. Patients getting MHD have up to three times per week dialysis treatment and vital sign information recorded, as well as frequent monthly laboratory testing, resulting in comprehensive electronic health record data that is appropriate for supporting prognostic models. As a result, the indices used in our scoring system were easily retrieved and time-updated, and more crucially, the indexes used in the scoring model were basic assessment items for all MHD patients, posing no additional load on them. We propose that the novel scoring model be widely used in most HD facilities for rapid and presumptive risk classification, as it is based on five clinical factors that are easily and frequently measured. If the overall score for MHD patients at a primary hospital exceeds 7 points and there are no hardware criteria for costly hematological testing, death is likely during the following 3 months. Our scoring model might be a valuable resource for referrals to tertiary institutions for additional tests and intervention, which can aid in the selection of therapy regimens and prognosis prediction.

Patients with ESKD, particularly those with MHD, were seen as having a chronic condition that requires well-established preventative health practices to have a longer life expectancy. However, the significant diversity in mortality is less well recognized: 20% of patients die within the first year after MHD onset, whereas 40% live for - over 5 years (Collins et al., 2013). Thus, recognizing which individuals are at high risk of dying shortly might help focus efforts to intervene on possibly controllable hazards, thereby improving the prognosis of MHD patients. However, insufficient evidence has been published to focus on the prognosis of the near-term survival of MHD patients. Li et al. established and validated a prediction model based on 852 incident HD patients and obtained an AUC of 0.758 (95%CI 0.677–0.836) for predicting the first 6 months of death (Li et al., 2022). Using the data from the Korean Society of Geriatric Nephrology (KSGN) database, Park et al. constructed the KSGN score based on the initially single characteristics for predicting 6-month mortality in 1751 incident HD patients and demonstrated that the KSGN score was outperformed for mortality prediction compared with two other commonly used score: Alberta Wick score, USRDS Thamer score (Thamer et al., 2015; Park et al., 2025; Wick et al., 2017). Similar results had also been made in other manuscripts for the near-term mortality in MHD patients (Xian et al., 2025; Goldstein et al., 2024; Do et al., 2023; Sakashita et al., 2024). In contrast to most of them, we applied the time-updated data from across dialysis treatments, considering that HD might impact multiple organ systems and acute clinical events could change a patient’s risk profile, therefore, biometric data routinely captured and updated within dialysis are predictive of adverse events. Moreover, different from these risk scores for predicting relatively longer mortality, understanding who is at highest risk of dying in the very near-term based on time-updated data could motivate focused efforts to intervene on potentially modifiable risks or, if not deemed modifiable, could guide decisions regarding patient-centered end-of-life care or de-escalation of burdensome medical therapies using shared decision-making approaches.

An interesting phenomenon emerged in this study that patients had a longer duration of dialysis (more than 1 year) had a significantly higher 3-month mortality compared with MHD patients with shorter dialysis vintage (26.8% vs. 10.6% in the training set, and 13.6% vs. 7.4% in the validation set, both P < 0.001). In previous studies, there were confusing and even contradictory results. On the one hand, the dialysis vintage was significantly associated with complications for MHD patients, such as cognitive frailty (Qin et al., 2025), fatigue (Qiao et al., 2024), acute ischemic stroke (Tong et al., 2024), and so on, but on the other, several studies had also demonstrated that the duration of dialysis did not relate with the prognosis of MHD patients (Sun et al., 2024; Qin et al., 2024; Erez et al., 2021). In addition, two studies showed that longer dialysis vintage was importantly related to a lower mortality rate for MHD patients (Huang et al., 2025; Xu et al., 2024). Our study added evidence that patients with relatively longer duration of HD experienced longer survival dates. This phenomenon might at least partly be explained by socioeconomic factors as well as psychological states during the initial stage of dialysis.

It is also necessary to take into account certain constraints. This study is first and foremost a retrospective analysis of MHD patients in a single area, this design means that we could not control for or even measure all potential confounding factors. Key clinical and socioeconomic variables—such as the precise burden of comorbidities (e.g., quantified by frailty scores), functional status, medication adherence, health literacy, and socioeconomic status—were not available in our database, which in turn, inherently carries the risk of selection bias and limits our ability to establish causality. Second, we were able to pinpoint significant predictor factors; however, these analyses are not mechanistic and do not pinpoint the cause of death or recommend suitable measures. Third, the model was still based on the most recent clinical assessment, but we incorporated time-updated data from different dialysis patient months. Therefore, the trajectory (i.e., changes over time) of biomarker values was not explicitly modeled. Fourthly, our prediction model’s capacity to stratify risks is relatively weak. Moreover, future researches should focus on prospective, multi-center external validation in geographically diverse populations to evaluate whether the implementation of this score in EHR systems, with automated calculation and clinical decision support alerts, actually improves patient outcomes, enhances goal-concordant care, or optimizes resource utilization. Finally, studies are needed to evaluate the impact of implementing this score on hard clinical outcomes, such as hospitalization rates and patient quality of life, to truly ascertain its value in clinical practice.

## Conclusion

In conclusion, we developed a prediction model based on easily accessible and time-updated factors for predicting near-term mortality using a large and heterogeneous dataset of patients receiving MHD. Our findings demonstrate that frequently available dialysis data may be used to create a reliable and fair tool. By identifying patients at elevated risk for near-term mortality, the score has the potential to inform clinical decision-making, such as triggering more frequent reviews, optimizing management of modifiable risk factors, or initiating conversations about patient goals and care preferences. Future research should focus on the prospective validation and implementation of this score in diverse clinical settings to evaluate its impact on patient outcomes and resource allocation.

## Data Availability

The original contributions presented in the study are included in the article/Supplementary Material, further inquiries can be directed to the corresponding author.
