# Supplementary material for: A time-updated scoring system derived from a nomogram to predict 3-month mortality in maintenance hemodialysis patients
Source: Front Aging. 2026 Jan 15;7:1716325. doi: 10.3389/fragi.2026.1716325 (PMC12852420; doi:10.3389/fragi.2026.1716325)
Supplement: Supplementary file 1 [file Table1.docx]

**Table S1 Univariate logistic regression analysis for 3-month mortality in the training cohort**

| Variables | VIF | Coefficients | OR (95%CI) | P value |
| --- | --- | --- | --- | --- |
| Age | 1.165 | 0.049 | 1.05 (1.04-1.06) | <0.001 |
| Gender, male | 1.056 | 0.006 | 1.01 (0.91-1.12) | 0.902 |
| Duration of dialysis | 1.187 | -0.196 | 0.82 (0.81-0.84) | <0.001 |
| Cause of ESKD | 1.031 |  |  |  |
| Chronic glomerulonephritis |  | -0.142 | 0.87 (0.75-1.00) | 0.051 |
| Diabetic nephropathy |  | 0.329 | 1.39 (1.21-1.60) | <0.001 |
| Hypertensive nephropathy |  | 0.136 | 1.15 (0.99-1.33) | 0.077 |
| Others or unknown |  | - | Ref. | - |
| Vascular access | 1.158 |  |  |  |
| AVF |  | - | Ref. | - |
| TCC |  | 0.261 | 1.30 (1.17-1.45) | <0.001 |
| Hemoglobin | 1.194 | -0.028 | 0.97 (0.96-0.98) | <0.001 |
| Lg (Platelets) | 1.036 | -0.785 | 0.46 (0.33-0.63) | <0.001 |
| Albumin | 1.343 | -0.169 | 0.84 (0.83-0.85) | <0.001 |
| Potassium | 1.258 | -0.630 | 0.53 (0.49-0.57) | <0.001 |
| Calcium | 1.075 | -0.389 | 0.68 (0.54-0.85) | 0.001 |
| Phosphorus | 1.304 | -0.621 | 0.54 (0.49-0.60) | <0.001 |
| Lg(iPTH) | 1.086 | -0.210 | 0.81 (0.72-0.91) | 0.001 |
| Lg(Ferritin) | 1.010 | -0.098 | 0.91 (0.80-1.03) | 0.139 |

ESKD, end-stage kidney disease, AVF, arteriovenous fistula, TCC, tunneled central venous catheter, iPTH, intact parathyroid hormone, VIF, variance inflation factor, OR, odds ratio, 95%CI, 95% confidence interval.

**Table S2 Multivariate logistic regression analysis for 3-month mortality in the training cohort**

| Variables | OR (95%CI) | P value |
| --- | --- | --- |
| Age | 1.04 (1.03-1.05) | <0.001 |
| Duration of dialysis | 0.87 (0.86-0.89) | <0.001 |
| Vascular access |  |  |
| AVF | Ref. | - |
| TCC | 1.71 (1.52-1.93) | <0.001 |
| Hemoglobin | 0.98 (0.97-0.99) | <0.001 |
| Albumin | 0.88 (0.87-0.90) | <0.001 |

AVF, arteriovenous fistula, TCC, tunneled central venous catheter, OR, odds ratio, 95%CI, 95% confidence interval.

**Table S3 Baseline characteristics of the risk groups in the training set**

| Characteristics | Low risk group | Moderate risk group | High risk group | P value |
| --- | --- | --- | --- | --- |
| N | 5627 | 8735 | 5373 |  |
| Age, years old | 49.4 ± 9.2 | 68.5 ± 9.5 | 71.4 ± 11.8 | <0.001 |
| Gender, male, n (%) | 3608 (64.1) | 5589 (64.0) | 3022 (56.2) | <0.001 |
| Duration of dialysis, years | 6.3 ± 2.2 | 5.6 ± 1.8 | 4.0 ± 1.6 | <0.001 |
| < 1 year, n (%) | 386 (6.9) | 295 (3.4) | 498(9.3) |  |
| 1-5 years, n (%) | 2226 (39.6) | 4388 (50.2) | 3509 (65.3) | <0.001 |
| 6-10 years, n (%) | 2014 (35.8) | 3048 (34.9) | 1058 (19.7) | <0.001 |
| > 10 years, n (%) | 1001 (17.8) | 1004 (11.5) | 308 (5.7) | <0.001 |
| Cause of ESKD, n (%) |  |  |  | 0.601 |
| Chronic glomerulonephritis | 2639 (46.9) | 2582 (29.6) | 1429 (26.6) | <0.001 |
| Diabetic nephropathy | 841 (14.9) | 2164 (24.8) | 1407 (26.2) | <0.001 |
| Hypertensive nephropathy | 872 (15.5) | 2132 (24.4) | 1034 (19.2) |  |
| Others or unknown | 1275 (22.7) | 1857 (21.3) | 1503 (28.0) | <0.001 |
| Vascular access, n (%) |  |  |  | <0.001 |
| AVF | 5216 (92.7) | 7123 (81.5) | 1970 (36.7) | 0.027 |
| TCC | 411 (7.3) | 1612 (8.5) | 3403 (63.3) | <0.001 |
| Laboratory results |  |  |  | <0.001 |
| Hemoglobin, g/L | 107.8 ± 17.6 | 106.9 ± 17.8 | 92.4 ± 15.0 | <0.001 |
| Platelets, × 10^9^/L | 176.9 ± 54.0 | 168.6 ± 54.3 | 169.5 ± 59.9 | <0.001 |
| Albumin, g/L | 40.4 ± 3.5 | 39.3 ± 3.3 | 34.5 ± 4.0 | <0.001 |
| Potassium, mmol/L | 4.9 ± 0.8 | 4.7 ± 0.8 | 4.4 ± 0.7 | <0.001 |
| Calcium, mmol/L | 2.3 ± 0.2 | 2.2 ± 0.2 | 2.2 ± 0.2 | <0.001 |
| Phosphorus, mmol/L | 1.9 ± 0.6 | 1.7 ± 0.5 | 1.5 ± 0.5 | <0.001 |
| iPTH, pg/mL | 507.7 ± 123.7 | 412.8 ± 102.9 | 354.9 ± 82.7 | <0.001 |
| Ferritin, ug/L | 187.9 ± 76.0 | 180.8 ± 56.8 | 169.2 ± 48.7 | 0.001 |
| Three-month death, n (%) | 111 (2.0) | 450 (5.2) | 1123 (20.9) | <0.001 |

ESKD, end-stage kidney disease, AVF, arteriovenous fistula, TCC, tunneled central venous catheter, iPTH, intact parathyroid hormone.

**Table S4 Logistic analysis of risk score for 3-month mortality in the training set**

| Subgroups | Case/N | OR (95%CI)^1^ | P value |
| --- | --- | --- | --- |
| All |  |  |  |
| Low risk | 111/5627 | Ref. |  |
| Moderate risk | 450/8735 | 2.61 (2.11-3.23) | <0.001 |
| High risk | 1123/5373 | 11.95 (9.71-14.69) | <0.001 |
| Age |  |  |  |
| < 60 years old |  |  |  |
| Low risk | 72/5423 | Ref. |  |
| Moderate risk | 95/781 | 3.55 (1.91-5.37) | <0.001 |
| High risk | 63/581 | 13.89 (9.85-18.04) | <0.001 |
| ≥ 60 years old |  |  |  |
| Low risk | 4/204 | Ref. |  |
| Moderate risk | 390/7954 | 1.59 (1.13-2.28) | <0.001 |
| High risk | 1060/4792 | 9.12 (7.80-16.98) | <0.001 |
| Gender |  |  |  |
| Male |  |  |  |
| Low risk | 78/3608 | Ref. |  |
| Moderate risk | 276/5589 | 2.28 (1.76-2.95) | <0.001 |
| High risk | 691/3022 | 12.42 (9.68-15.95) | <0.001 |
| Female |  |  |  |
| Low risk | 33/2019 | Ref. |  |
| Moderate risk | 174/3146 | 3.39 (2.32-4.95) | <0.001 |
| High risk | 432/2351 | 11.51 (7.96-16.65) | <0.001 |
| Cause of ESKD |  |  |  |
| Chronic glomerulonephritis |  |  |  |
| Low risk | 30/2639 | Ref. |  |
| Moderate risk | 99/2582 | 3.40 (2.24-5.14) | <0.001 |
| High risk | 342/1429 | 26.02 (17.59-38.50) | <0.001 |
| Diabetic nephropathy |  |  |  |
| Low risk | 33/841 | Ref. |  |
| Moderate risk | 135/2164 | 1.58 (1.07-2.34) | 0.022 |
| High risk | 307/1407 | 6.07 (4.14-8.90) | <0.001 |
| Hypertensive nephropathy |  |  |  |
| Low risk | 16/872 | Ref. |  |
| Moderate risk | 109/2132 | 2.72 (1.59-4.64) | <0.001 |
| High risk | 238/1034 | 14.25 (8.40-24.18) | <0.001 |
| Others or unknown |  |  |  |
| Low risk | 32/1275 | Ref. |  |
| Moderate risk | 107/1857 | 2.23 (1.49-3.34) | <0.001 |
| High risk | 236/1503 | 6.10 (4.12-9.04) | <0.001 |

ESKD, end-stage kidney disease, OR, odds ratio, 95%CI, 95% confidence index.

^1^Model was adjusted for gender, cause of ESKD, laboratory results (except for serum albumin and hemoglobin).

**Table S5 Logistic analysis of risk score for 3-month mortality in the test set**

| Subgroups | Case/N | OR (95%CI) | P value |
| --- | --- | --- | --- |
| All |  |  |  |
| Low risk | 153/5138 | Ref. |  |
| Moderate risk | 548/6164 | 3.08 (2.56-3.71) | <0.001 |
| High risk | 969/3963 | 8.55 (7.08-10.33) | <0.001 |
| Age |  |  |  |
| < 60 years old |  |  |  |
| Low risk | 118/4705 | Ref. |  |
| Moderate risk | 67/706 | 3.19 (2.29-4.46) | <0.001 |
| High risk | 67/494 | 4.21 (2.97-6.06) | <0.001 |
| ≥ 60 years old |  |  |  |
| Low risk | 25/433 | Ref. |  |
| Moderate risk | 491/5458 | 2.36 (2.04-2.92) | <0.001 |
| High risk | 902/3469 | 10.25 (7.26-14.66) | <0.001 |
| Gender |  |  |  |
| Male |  |  |  |
| Low risk | 121/3338 | Ref. |  |
| Moderate risk | 374/3940 | 2.73 (2.21-3.38) | <0.001 |
| High risk | 608/2328 | 7.78 (6.24-9.69) | <0.001 |
| Female |  |  |  |
| Low risk | 32/1800 | Ref. |  |
| Moderate risk | 174/2224 | 4.43 (3.01-6.50) | <0.001 |
| High risk | 361/1635 | 11.58 (7.89-17.00) | <0.001 |
| Cause of ESKD |  |  |  |
| Chronic glomerulonephritis |  |  |  |
| Low risk | 34/2043 | Ref. |  |
| Moderate risk | 125/1713 | 4.62 (3.13-6.82) | <0.001 |
| High risk | 217/974 | 13.92 (9.35-20.72) | <0.001 |
| Diabetic nephropathy |  |  |  |
| Low risk | 57/909 | Ref. |  |
| Moderate risk | 191/1612 | 1.97 (1.44-2.69) | <0.001 |
| High risk | 307/1105 | 4.90 (3.57-6.74) | <0.001 |
| Hypertensive nephropathy |  |  |  |
| Low risk | 29/869 | Ref. |  |
| Moderate risk | 110/1419 | 2.23 (1.46-3.41) | <0.001 |
| High risk | 223/809 | 8.58 (5.62-13.11) | <0.001 |
| Others or unknown |  |  |  |
| Low risk | 33/1317 | Ref. |  |
| Moderate risk | 122/1420 | 3.54 (2.38-5.25) | <0.001 |
| High risk | 222/1075 | 7.99 (5.35-11.93) | <0.001 |

ESKD, end-stage kidney disease, OR, odds ratio, 95%CI, 95% confidence index.

^1^Model was adjusted for gender, cause of ESKD, laboratory results (except for serum albumin and hemoglobin).

**Table S6 Comparison the performance of our risk score with previous risk scores**

|  | AUC (95%CI) | Sensitivity (95%CI) | Specificity (95%CI) | Brier score(95%CI) |
| --- | --- | --- | --- | --- |
| In the training set |  |  |  |  |
| Risk score | 0.71 (0.69-0.75) | 0.61 (0.57-0.68) | 0.73 (0.70-0.74) | 0.18 (0.16-0.19) |
| Tharmer score | 0.67 (0.62-0.71) | 0.76 (0.61-0.82) | 0.58 (0.53-0.62) | 0.19 (0.17-0.20) |
| KSGN score | 0.68 (0.63-0.72) | 0.57 (0.51-0.63) | 0.77 (0.72-0.79) | 0.18 (0.17-0.20) |
| In the validation set |  |  |  |  |
| Risk score | 0.70 (0.66-0.75) | 0.68 (0.60-0.81) | 0.70 (0.61-0.80) | 0.15 (0.13-0.16) |
| Tharmer score | 0.71 (0.67-0.76) | 0.73 (0.64-0.85) | 0.69 (0.60-0.78) | 0.15 (0.13-0.17) |
| KSGN score | 0.64 (0.59-0.68) | 0.56 (0.53-0.64) | 0.78 (0.75-0.81) | 0.17 (0.15-0.19) |

We only included patients with age more than 67 years old and the HD duration less than 1 year.

AUC, area under the curve, 95%CI, 95% confidence interval.


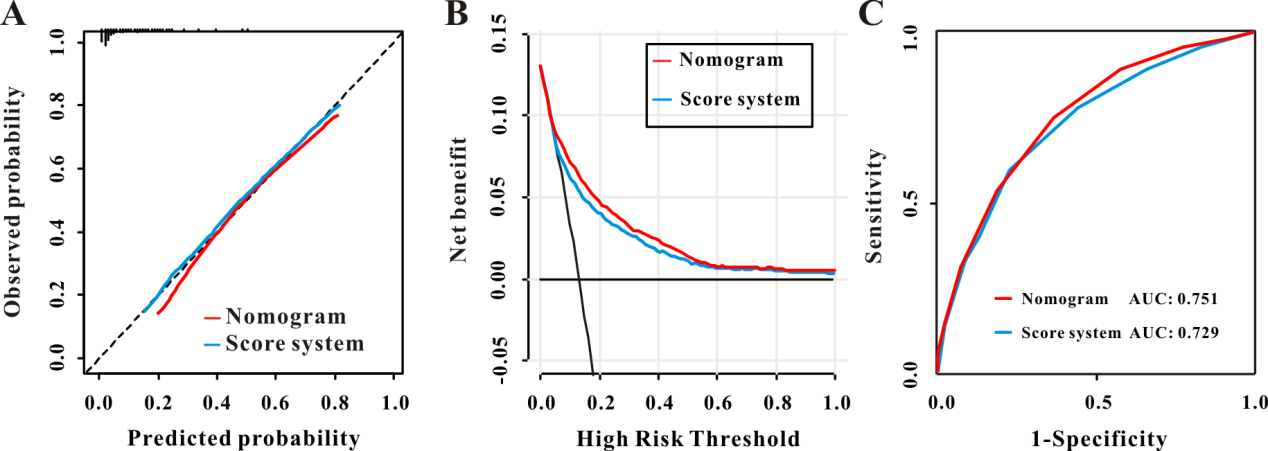
**Figure S1** The calidation plot (**A**), decision curve analysis (**B**), and the receiver operator characteristic curve (**C**) of the nomogram and the score system for 3-month mortality in the validation set.


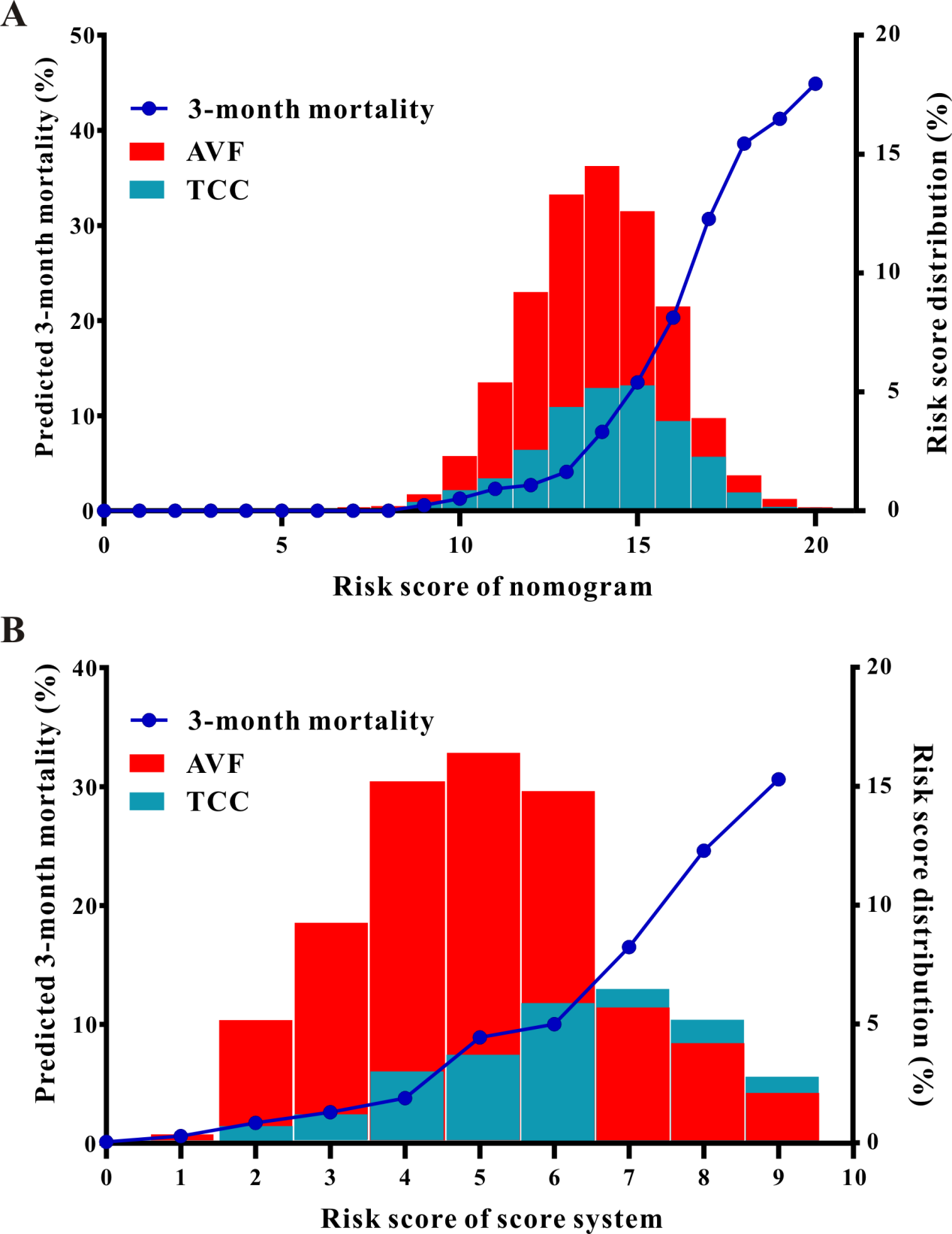


**Figure S2** Scores calculated with the nomogram (**A**) and the score system (**B**) were plotted on the x-axis of this figure, enabling the inference of predicted 3-month mortality by correlating each score with the appropriate plotted lines. The histogram depicts the risk score distribution of the validation as an example of the distribution of risk scores across the patient population (each bar represents the proportion of patients in the cohort that was assigned that specific score).
